# Assessment of Food Environment in Public Facilities of Urban Municipalities in Nepal: A Baseline Study for Municipal-Level Healthy Food Procurement and Service Policy Development

**DOI:** 10.31729/jnma.v63i2091.9233

**Published:** 2025-11-30

**Authors:** Namuna Shrestha, Priza Pradhananga, Kalpana Chaudhary, Nicole Ide, Nabin Adhikari, Archana Shrestha

**Affiliations:** 1 Department of Public Health and Community Programs, Dhulikhel Hospital, Dhulikhel, Nepal; 2 Institute for Implementation Science and Health, Kathmandu, Nepal; 3 Resolve to Save Lives, Virginia, USA; 4 College of Health and Agricultural Sciences, School of Medicine, University College Dublin, Dublin, Ireland; 5 Department of Public Health, Kathmandu University School of Medical Sciences, Dhulikhel, Nepal

**Keywords:** *feeding behavior*, *nutrition policy*, *public facilities*

## Abstract

**Introduction:**

Food environments in public facilities, particularly schools, influence dietary behaviours yet often lack healthy eating standards, particularly in low-resource contexts, contributing to the rising burden of non-communicable diseases. This study assessed the current food environment in public facilities within four urban municipalities of Bagmati Province, Nepal, as a baseline survey for developing a healthy food procurement and service policy.

**Methods:**

We conducted a cross-sectional study from May to December 2022 in 355 public facilities, including schools, healthcare facilities, and care homes. We collected data on food procurement, food availability, cooking practices, food advertisement, and hygiene through inperson interviews and observations, and analyzed using STATA 17.

**Results:**

Out of 355 public facilities, 349 (98.30%) were schools. Vegetables were available as a healthy option with a median of 12 items per week (IQR: 9-16), followed by fruits 1 (IQR: 0-6) and whole grains 0 (IQR: 0-1). Among unhealthy options, refined grains were available with a median of 14 (IQR: 1021), followed by deep-fried foods 2 (IQR: 0-6) and sugar-sweetened beverages 0 (IQR: 0-1). There were 22 (6.19%) facilities with measures to limit junk food, while 95 (26.76%) had monitoring mechanisms, mainly by facility committees.

**Conclusions:**

The findings highlight a concerning imbalance in public facility canteens, where unhealthy food options are more prevalent. Facilities also have limited canteen monitoring mechanisms and initiatives for promoting a healthy food environment.

## INTRODUCTION

Unhealthy food environments in public facilities contribute to rising rates of obesity and non-communicable diseases (NCDs), accounting for over 71% of total deaths.^1-3^ Consumption of foods high in sugar, salt, and saturated fats, along with widespread marketing of ultra-processed foods, often hinders healthy choices.^4,5^ Nepal’s rising food prices and demand for convenience foods pose additional challenges. ^5^

This is a cross-sectional study as a part of a comprehensive program to develop, implement, and evaluate a municipal healthy food procurement and service policy in Nepal. We conducted this study from May to December 2022 in four urban municipalities of Kathmandu Valley, Nepal (Budhanilkantha, Madhyapur Thimi, Tokha, and Kageshwori Manohara). We selected these the current food environment in public facilities is also limited, particularly at the municipal level. This study aimed to assess the food environment in public facilities within four municipalities in Bagmati Province, Nepal, to inform policy development and provide a baseline for policy evaluation.

## METHODS

Although the World Health Organization (WHO) recommends healthy food procurement and service policies through its Action Framework for public food procurement and identifies ‘Best Buys’ as cost-effective interventions to prevent NCDs, no such policies are in place in Nepal to regulate the purchase and availability of healthy foods in public facilities.^6,7^ Evidence on municipalities using purposive sampling following rapid assessment of 10 municipalities. The assessment used the following criteria, including municipal expressed interest and commitment; and readiness to engage in policy development, implementation and evaluation. Ethical approval was obtained from the Nepal Health Research Council (Ref no. 454/2020P) and the Institutional Review Board (Ref no. 140/20).

The municipalities’ administrative offices provided a comprehensive list of all public facilities. From the list, we contacted all facilities for eligibility screening through telephonic interviews. The facility inclusion criteria were (a) government schools, private schools, government worksites, government hospitals, government care homes, and government correctional facilities; (b) facilities with their own canteens. Facilities that met these criteria were invited to participate.

Of the 418 screened facilities, 355 met the eligibility criteria and were enrolled. We adopted a census approach, including all eligible facilities. We then visited the eligible facilities with the official support letter from the municipality. We first contacted the head/representative of the facilities; briefed about the study and its objectives; and requested to visit the canteen. Canteen managers/staff involved in food procurement and services who were aged 15-59 and able to communicate verbally were invited as participants. All the participants who agreed to provide consent and participated in the study were older than 18 years. We conducted in-person interviews and observations at each site. We obtained written informed consent to ensure voluntary participation and confidentiality. Trained data collectors used electronic questionnaires on tablets via Kobo Toolbox software. The tool was developed de novo based on comprehensive literature review and consultations with public health and nutrition experts. It was pretested with 10% of the sample outside the study site including 14 schools, 5 hospitals, 3 government offices and 13 correctional facilities. The tools were then revised accordingly. The Cronbach’s alpha coefficient for the food availability section (frequency of food items available per week) was 0.66, indicating acceptable internal consistency whereas other sections on facility and canteen characteristics, and procurement processes were refined after pretesting for clarity and relevance. An observational checklist covered food advertisement, cooking practices, and hygiene which were also developed through literature review and expert input. Enumerators received training and conducted mock observations to ensure consistency and reduce observer bias.

We analyzed data using STATA 17. We presented categorical data as frequency and percentage, and numerical data using median (interquartile range, IQR). We categorized food availability, comprising 68 food items, into 15 food groups and segmented them into positively (healthy) and negatively (unhealthy) scored components following the Prime Diet Quality Score.^8^ We calculated the median (IQR) of different food items available per week for each food group.

## RESULTS

Out of 355 public facilities, 349 (98.30%) were schools, with 63.89% secondary level and 86.53% being private. The median number of employees was 29 (IQR: 13-40), students 300 (IQR:91-500), canteen staff 3 (IQR:2-4), and daily customer 100 (IQR:60-200) (Table 1).

**Table 1:** Characteristics of public facilities of urban municipalities (n=355).

Characteristics	Budhanilkantha (n=107)	Tokha (n=83)	Kageshwori (n=85)	Thimi (n=80)
**Type of facility n(%)**				
School	107(100)	80(96.38)	85(100)	77(96.25)
Hospital	0(0)	3(3.61)	0(0)	0(0)
Care home	0(0)	0(0)	0(0)	3(3.75)
Level of school* n(%)				
Montessori	27(25.23)	20(25.00)	47(55.29)	46(59.74)
Primary level	12(11.21)	6(7.50)	8(9.41)	9(11.68)
Secondary level	70 (65.42)	56(70.00)	57(67.05)	40(51.94)
Higher secondary school	5(4.67)	5(6.25)	1(1.17)	7(9.09)
Hostel	13(12.15)	3(3.75)	0(0)	1(1.29)
**Type of school n(%)**				
Government	6(5.61)	1(1.25)	12(14.11)	17(22.07)
Private	95(88.79)	79(98.75)	68(80.00)	60(77.92)
Community	6(5.61)	0(0)	5(5.88)	-
Number of employees, Median (IQR)	25.00(13-38)	30.00(18-45)	30.00(13-44)	27.00(10-40)
Number of students, Median (IQR)	250.00(87-482)	328.00(150-450)	350.00(82-600)	300.00(65-430)
Number of staff in canteen, Median (IQR)	3.00(2-4)	3.00(2-4)	3.00(2-4)	2.00(2-3)
Number of customers in canteen per day, Median (IQR)	100.00(60-200)	138.00(60-250)	120.00(70-250)	100.00(50-187)

* Multiple response (n=349)

Canteens offered both free and purchased foods. There were 340 (95.77%) of canteens that had food available for purchase by customers, and 52 (14.64%) offered food free of cost, funded by sources like Mid-Day Meal programs, donations, and support from municipalities and organizations. Canteens operated through mutual agreements between the facility and canteen were 240 (67.60%) and funded by sales revenue in 261 (73.72%) facilities. Canteens operated six days per week on average, and 353 (99.40%) prepared meals in-house. Facility administration primarily decided the food selection in 267 (75.21%) facilities, with 114 (75.21%) of canteen operators having some input. Similarly, the administration developed the menu in 200 (85.83%) of facilities with minimal input from 20 (8.58%) nutritionists/dietitians. There was a set menu in 230 (64.78%) of canteens, with 225 (97.82%) of them consistently adhering to it (Table 2).

**Table 2:** Characteristics of canteen operation and service in public facilities of urban municipalities (n=355).

Characteristics	Budhanilkantha (n=107)	Tokha (n=83)	Kageshwori (n=85)	Thimi (n=80)
Process of food distribution* n(%)				
Free of cost	22(20.56)	4(4.81)	12(14.11)	14(17.50)
Purchase	102(95.33)	81(97.59)	82(96.47)	75(93.75)
Types of meals* n(%)				
Breakfast	30(28.04)	20(24.01)	23(27.05)	17(25.35)
Lunch	75(70.09)	57(68.67)	42(49.41)	42(52.50)
Snacks	102(95.33)	83(100.00)	85(100.00)	78(97.50)
Dinner	17(15.89)	11(13.25)	3(3.52)	3(3.75)
**Process of canteen operation n(%)**				
Bidding process	2(1.87)	6(7.22)	11(12.94)	2(2.50)
Mutual agreement between facility and canteen operator	67(62.62)	68(81.92)	51(60.00)	54(67.50)
Operated by facility	38(35.51)	9(10.84)	23(27.05)	24(30.00)
Canteen operation (months), Median (IQR)	60(12-120)	60(18-20)	48(12-90)	96(24-41)
Days of service (Mean±SD)	6.1±0.36	6.17±0.60	6.01±0.70	6.2±0.80
Food cooked in canteen n(%)	105(98.10)	83(100)	85(100)	80(100.00)
Source of fund* n(%)		6.2±0.80		
Revenue generated in canteen	69(64.49)	74(89.15)	62(72.94)	56(70.90)
Government fund	8(7.48)	1(1.20)	10(11.76)	14(17.70)
Welfare fund of facility	33(30.84)	8(9.63)	21(24.70)	18(22.50)
Supported by other aids	3(2.80)	1(1.20)	-	1(1.25)
Decision maker for food selection* n(%)				
Canteen operator	43(40.19)	30(36.14)	21(24.70)	20(25.00)
Administration of facility	75(70.09)	58(69.87)	71(83.52)	63(78.75)
Jointly with customer	3(2.80)	6(7.22)	9(10.58)	11(13.75)
Others	6(5.61)	-	-	1(1.25)
Availability of set menu n(%)	73(68.22)	42(50.60)	59(69.41)	56(70.00)
Adhering to a set menu(n=230) n(%)	71(97.26)	40(95.23)	59(100.00)	55(98.21)
Menu developer* (n=230) n(%)				
Administrative section	58(76.32)	37(88.09)	55(93.22)	50(89.28)
Canteen owner	32(42.11)	3(7.14)	3(5.08)	7(12.50)
Nutritionist/Dietician	4(5.26)	7(16.66)	4(6.77)	5(8.92)
Others	3(3.95)	-	2(3.38)	-

* Multiple response

There were 347 (97.74%) facilities that sourced food from local markets, such as rice, vegetables, fruits, meat, and dairy. Local farmers provided fresh items such as vegetables and pulses. There were 353 (99.43%) facilities that reported having easy access to markets. There were 22 (6.19%) facilities that had general guidelines to limit junk food. About 95 (26.76%) facilities have canteen monitoring mechanisms for basic food safety measures or to measure informal efforts to increase the availability of healthy foods. Monitoring was led by facility committees in 55 (57.89%) facilities and government agencies in 36 (37.89%) facilities. Penalties included fines in 27(28.42%) facilities, canteen bans in 11(11.57%), and warnings in 42 (44.21%), with 3(3.15%) facilities reporting no penalties (Table 3).

The canteens offered the healthy option as vegetables (cooked or fresh), with a median of 12 items served per week (IQR:9-16). Beans, peas, and soy products were available with a median of 9 (IQR:6-12) per week, followed by fruits 1 (IQR: 0-6), poultry 2 (IQR: 0-3), low-fat dairy 0 (IQR: 0-6), nuts and seeds 0 (IQR: 0-2), and whole grains 0 (IQR: 0-1) per week. Unhealthy food options like refined grains had a median of 14 items per week (IQR:10-21) followed by deep-fried foods at 2 (IQR: 0-6) per week (Table 4).

**Table 3:** Food procurement practice and monitoring mechanism in school canteens (n=355).

Characteristics	Budhanilkantha n=107	Tokha n=83	Kageshwori n=85	Thimi n=80	Total n=355(100)
**Source of purchasing food n(%)**					
Local market/supermarket	107(100.00)	79(95.18)	84(98.82)	77(96.25)	347 (97.74)
Local farmer	23(21.50)	18(21.68)	9(10.58)	15(18.75)	65 (18.30)
Easy access of food markets	107(100.00)	83(100.00)	85(100.00)	79(98.75)	353 (99.43)
Existing food and nutrition policy	-	-	-	-	-
Any healthy initiatives followed	21(19.63)	-	-	1(1.25)	22 (6.19)
Availability of monitoring mechanism	49(45.79)	12(14.45)	6(7.05)	28(35.00)	95 (26.76)
Monitoring in last one year	43(87.75)	9(75.00)	4(66.66)	14(50.00)	70 (73.68)
**Frequency of monitoring in a year, Median (IQR) (Days)**	20(3-200)	1(1-1)	4.50(1.75-5.75)	2(1-6.50)	9.6±11.00
Responsible body for monitoring (n=95)* n(%)					
Management committee within facility	32(65.31)	4(33.33)	3(50.00)	16(57.14)	55 (57.89)
Government agencies	15(30.61)	7(58.33)	2(33.33)	12(42.85)	36 (37.89)
Student union/Staff union within facility	3(6.12)	-	-	-	3 (3.15)
Others	2(4.08)	2(16.66)	1(16.66)	6(11.57)	11 (11.57)
Penalties for non-compliance (fines) (n=95)* n(%)					
Financial	2(4.08)	9(75.00)	3(50.00)	13(46.42)	27 (28.42)
Ban on running canteen	1(2.04)	3(25.00)	-	7(25.00)	11 (11.57)
Warning	11(22.45)	8(66.66)	5(83.33)	18(64.28)	42 (44.21)
None	3(6.12)	-	-	-	3 (3.15)
Don’t know	37(75.51)	1(8.33)	1(16.66)	9(32.10)	48 (50.52)

* Multiple response

**Table 4:** Number of healthy and unhealthy food items available weekly in canteens (n=355).

Food groups	**Median (IQR) of food available in canteen**			
Healthy foods	Budhanilkantha	Tokha	Kageshwori	Thimi
Vegetables	9(4-13)	14(11-18)	12(9-18)	12(8-17)
Fruits	0(0-3)	3(0-6)	2(0-6)	3(1-6)
Beans, peas, and soy products	8(6-10)	10(8-14)	9(6-14)	8(5-11)
Nuts and seeds	0(0-0)	0(0-1)	0(0-3)	0(0-3)
Poultry	2(1-4)	2(1-4)	2(0-3)	2(0-2)
Fish	1(1-1)	1(1-1)	1(1-1)	1(1-1)
Whole grain	0(0-1)	1(0-1)	0(0-1)	0(0-1)
Low-fat dairy	0(0-6)	0(0-6)	0(0-6)	1(0-6)
**Unhealthy foods**					
Red meat	0(0-0)	0(0-0)	0(0-0)	0(0-0)
Processed meat	1(1-1)	1(1-1)	1(1-1)	1(1-1)
Refined grains	15(11-23)	15(10-24)	14(10-20)	13.5(9-17)
Sugar-sweetened beverages	0(0-6)	0(0-0)	0(0-0)	0(0-0)
Sweets and ice cream	0(0-0)	0(0-0)	0(0-0)	0(0-0)
Deep fried food	3(1-6)	3(1-6)	2(1-7)	1(0-4)
Others	13(12-24)	13(7-19)	13(10-18)	12(6-14)

**Table 5:** On-site observation of canteen environment, cooking practices and hygiene (n=355).

Characteristics	Budhanilkantha (n=107)	Tokha (n=83)	Kageshwori (n=85)	Thimi (n=80)
**Advertisement and promotion activities n(%)**				
Availability of logos	6(5.60)	2(2.40)	4(4.70)	-
Availability of promotion activities	11(10.28)	-	1(1.17)	3(3.75)
Types of oil used for cooking* n(%)				
Dalda	1(0.93)	-	-	2(2.50)
Vegetable oil	107(100.00)	83(100.00)	85(100.00)	79(98.75)
Ghee/Butter	1(0.93)	-	3(3.52)	3(3.75)
Types of vegetable oil* n(%)				
Sunflower oil	91(85.05)	68(81.92)	64(75.29)	60(75.9)
Soybean oil	27(25.23)	24(28.91)	26(30.58)	25(31.60)
Mustard oil	3(2.80)	4(4.81)	6(7.05)	9(11.40)
Others	1(0.93)	0	2(2.35)	1(1.00)
Reuse of cooking oil	23(21.49)	16(19.27)	10(11.76)	17(21.25)
**Type of salt n(%)**				
Iodized salt	107(100.00)	81(97.59)	85(100.00)	80(100.00)
Availability of handwashing facility n(%)	81(75.70)	79(95.18)	80(94.11)	71(88.75)
Availability of sugar on dining table n(%)	9(8.41)	4(4.81)	3(3.52)	-
Availability of condiments on dining table n(%)	6(5.60)	7(8.43)	5(5.88)	1(1.25)
Cleanliness of plates and utensils n(%)	99(92.52)	77(92.77)	74(87.05)	79(98.75)
Use of apron by staff/cook n(%)	42(39.25)	44(53.01)	51(60.00)	39(48.75)
Use of hairnets by staff/cook n(%)	17(15.88)	12(14.45)	14(16.47)	13(16.25)
Use of masks by staff/cook n(%)	25(23.36)	4(4.81)	14(16.47)	13(16.25)
Display of healthy eating posters n(%)	-	4(4.81)	2(2.35)	-
Availability of salt on the dining table n(%)	9(8.41)	1(1.20)	3(3.52)	1(1.25)
Cleanliness of the kitchen and dining area n(%)	90(84.11)	76(91.56)	79(92.94)	68(85.00)
Availability of drinking water n(%)	98(91.58)	82(98.79)	82(96.47)	77(96.25)
Availability of separate mechanism for waste disposal n(%)	80(74.76)	77(92.77)	79(92.94)	66(82.50)

* Multiple Response

There were 12(3.38%) facilities that displayed logos of unhealthy foods, and 15 (4.22%) facilities had promotion activities, including pricing specials and discounts. Display of healthy eating posters and condiments on dining tables was seen in 6 (1.69%) facilities. Staff used vegetable oil in 354 (99.71%) facilities, primarily sunflower oil in 283 (79.94%) facilities. However, 66 (18.59%) facilities reused oil. There were 353 (99.43%) facilities using iodized salt. Hygiene practices were generally good, with 311 (87.60%) having availability of handwashing facilities and 339 (95.49%) facilities had drinking water. However, staff and cooks wore aprons in 176 (49.57%) facilities and hairnets/masks in 56 (15.77%) facilities. There were 313 (85.16%) facilities with waste disposal systems (Table 5).

## DISCUSSION

This study assessed the food environment in 355 public facilities of four urban municipalities in Nepal, with most being schools (98.30%). All facilities lacked formal policies to regulate food procurement and service. Only 6.19% had measures for limiting junk foods, and 26.76% monitored food safety and hygiene. Vegetables and legumes were commonly available, but fruits, whole grains, low-fat dairy, and nuts were limited. Refined grains were the most frequent unhealthy food option, followed by deep-fried items. The reuse of cooking oil was found in 18.59% of canteens, and a few displayed messages promoting healthy eating.

Most canteens prepare meals in-house, which allows greater control over food quality. However, because canteens often rely on revenue from food sales (73.72%), some may likely be hesitant to change their menus, fearing poor acceptance by students, which may limit their willingness to provide healthier and balanced meals. In higher-income settings, external funding from programs such as national universal free school meals often supports school canteens, reducing their need to rely on less healthy, more profitable food items to sustain operations. ^9,10^ The Government of Nepal has implemented the Mid-Day Meal Program (MDM) for government schools up to grade 3, aiming to improve enrolment, attendance and retention, and students' nutritional status.^11^ While the program has improved food access, schools face challenges in offering healthier food due to the limited per-student budget (NRs. 15). In this study, only 14.64% of facilities provided free meals, primarily through MDM. Most public facilities in this study sourced their food from local markets and farmers, with fresh vegetables and pulses being a prominent part of the supply chain. This practice is beneficial from a local economic perspective, but is often hindered by inconsistent food quality and safety standards.^12^ The lack of policies that establish nutrition criteria prioritizing availability, purchase, and quality of these healthy options further limits their consistent provision.

In Nepal, the sales and demand of unhealthy foods are increasing, and they are widely available in public facilities that commonly serve vulnerable populations, including children. ^1,5^ The Government of Nepal has recognized nutrition and food security as national priorities, and has introduced specific recommendations in the Nepal Food Based Dietary Guideline, 2012 and the School Health and Nutrition Strategy, 2006.^13,14^ While some facilities in this study have initiated practices to limit junk food within their premises, efforts are often informal and inconsistent, and no specific policies are in place to regulate the purchase and availability of healthier food. The lack of clear and sustainable policy harms food availability and consumption, allowing public facility food vendors to supply food items that increase the risk of diet-related diseases.^15^ Aligning with international recommendations, such as the WHO's Action Framework and WHO’s Best Buys for NCD prevention, can guide the development of cost-effective, structured policies that prioritize health and nutrition in public settings.^6,7^

A major finding of this study was the limited availability of healthy foods in canteens. Whereas, the canteens had a relatively good presence of vegetables (median 12, IQR:9-16). Healthy options such as fruits, whole grains, low-fat dairy, and fish were limited, while calorie-dense, less nutritious options like refined grains (median 14, IQR:10-21) and deep-fried foods (median 2, IQR: 0-6) were much more common. Sugary drinks were less available. These findings are consistent with previous studies in Malaysia, the United States, and Turkey, which have also observed that schools and public canteens often prioritize high-calorie, low-nutrient foods over healthier options.^16^'^19^ This trend may be closely linked to poor dietary choices among students.^17,20-22^ Research shows that when students have greater access to a la carte food options, they tend to consume more low-nutrient, energy-dense foods, like sugar-sweetened beverages, and fewer fruits and vegetables, which may increase students' intake of fats and sugar, resulting in poor energy balance and risk of excessive weight gain.^16,17,23^

Although a majority of sites used vegetable oils, rather than oils/fats higher in saturated or trans fats, 18.59% of facilities reported reusing leftover oil. While this is lower compared to the 80% found in the Indian study, it should be limited further as it produces harmful compounds linked to cardiovascular risk. Procurement and service policies can introduce standards to ensure healthy cooking practices, such as banning refrying, deep frying, or setting maximum limits for the overall quantity of added fats, oils, sugar, and salt. ^24-26^

Hygiene practices in canteens were generally adequate, with access to clean drinking water and handwashing facilities. However, few kitchen staff used safety measures like aprons and hairnets, suggesting gaps in food safety practices. This finding is consistent with a study in Ethiopia where poor hygiene in public canteens contributed to foodborne illnesses. ^27^ Good hygiene practice by food handlers is crucial, as the transmission of foodborne diseases is either through poor personal hygiene or cross-contamination.^28,29^ Proper handwashing among food handlers can substantially reduce the risk of diarrheal diseases in childcare facilities.^28^’^30^

Only 26.76% of facilities in this study had a basic canteen monitoring system, mostly on hygiene and sanitation, with few on healthy food availability. Although not policy-driven, these can be leveraged to expand the scope for monitoring future policy implementation, as monitoring is considered an integral part of effective policy implementation.^31,32^ Commitment from government and related institutions is essential to ensure policies are effective and sustainable.^33^

To the best of our knowledge, this study is the first assessment of the food environment in urban public facilities of Nepal, providing baseline insights into food procurement, availability, and hygiene practices. These findings can guide policy development to improve dietary habits in public settings. However, it has certain limitations. Its cross-sectional design limits assessment of longterm trends, and its limited geographic scope may not reflect the broader national context, especially in rural areas. Nonetheless, the study plays a critical foundation for addressing food environment gaps and developing a healthy food procurement policy in Nepal.

To encourage healthy food environments, the Government of Nepal should develop and implement a national-level food procurement and service policy framework that promotes healthier choices while supporting local governments to adapt the policy to their contexts. Strengthening programs such as offering healthy choices in the Mid-Day Meal Program, training canteen staff on healthy food preparation, and educational programs to students and parents can further ensure long-term improvements in dietary habits and health outcomes across public facilities.

## CONCLUSION

This study highlights significant gaps in the availability of healthy food and an absence of standardized policies for regulating food procurement and services in public facilities of Kathmandu Valley. Therefore, it suggests the need for policy-level actions to strengthen healthy food environments in similar urban settings.

## Data Availability

The data are available from the corresponding author upon reasonable request
